# A New Process for Purifying the Waters Supplied to the Metropolis by the Existing Water Companies

**Published:** 1841-10

**Authors:** 


Art. XV.—*A new Process for Purifying the Waters supplied to the Metropolis by the existing Water Companies.* By Thomas Clark, Professor of Chemistry in the University of Aberdeen.—*London*, 1841. 8vo, pp. 16.

In this little pamphlet we have detailed the particulars of an invention,—for so we may term this new application of a chemical fact already known—which promises to be of vast importance to the inhabitants of this metropolis, and, indeed, to the inhabitants of every city supplied with water artificially from a common source or sources. Professor Clark has obtained a patent for the invention; and, as very little preparation is necessary to put it in operation, we hope that we may look forward to see the process applied, ere long, to the cleansing of the filthy fluids we are all compelled to drink in this great city. That it will be eventually adopted we entertain no doubt; although it is not unlikely that it may at first meet with neglect and even with opposition.

The process employed by Dr. Clark is one no less simple and beautiful in its simplicity, then it is strictly scientific; its admirable applicability to the end in view, and its perfect efficiency will be self-evident to any one who considers the subject. We ourselves have had the advantage of witnessing some of Professor Clark's experiments, and the result was in the highest degree satisfactory. The following extract explains very clearly, and in familiar language, the theory of the process:

"To understand the nature of the process, it will be necessary to advert, in a general way, to a few long-known chemical properties of the familiar substance chalk; for chalk at once forms the bulk of the chemical impurity that the process will separate from water, and is the material whence the ingredient for effecting the separation will be obtained.

"In water, chalk is almost or altogether insoluble; but it may be rendered soluble by either of two processes of a very opposite kind. When burned, as in a kiln, chalk loses weight. If dry and pure, only nine ounces will remain out of a pound of sixteen ounces. These nine ounces will be soluble in water, but they will require not less than forty gallons of water for entire solution. Burnt chalk is called caustic lime, and water holding caustic lime in solution is called lime-water. The solution thus named is perfectly clear and colourless. The seven ounces lost by a pound of chalk on being burned, consists of carbonic acid gas—that gas which, being dissolved under compression by water, forms what is called soda-water.'

"The other mode of rendering chalk soluble in water is nearly the reverse. In the former mode, a pound of pure chalk becomes dissolved in water in consequence of losing seven ounces of carbonic acid. To dissolve in the second mode, not only must the pound of chalk not lose the seven ounces of carbonic acid that it contains, but it must combine with seven additional ounces of that acid. In such a state of combination, chalk exists in the waters of London—dissolved, invisible, and colourless, like salt in water. A pound of chalk, dissolved in 500 gallons of water by seven ounces of carbonic acid, would form a solution not sensibly different, in ordinary use, from the filtered water of the Thames, in the average state of that river. Chalk, which chemists call carbonate of lime, becomes what they call bicarbonate of lime when it is dissolved in water by carbonic acid.

"Any lime-water may be mixed with another, and any solution of bicarbonate of lime with another, without any change being produced:- the clearness of the mixed solutions would be undisturbed. Not so, however, if lime-water be mixed with a solution of bicarbonate of lime: very soon a haziness appears; this deepens into a whiteness, and the mixture soon acquires the appearance of a well-mixed whitewash. When the white matter ceases to be produced, it subsides, and in process of time leaves the water above perfectly clear. The subsided matter is nothing but chalk. What occurs in this operation will be understood, if we suppose that one pound of chalk, after being burned to nine ounces of caustic lime, is dissolved, so as to form forty gallons of lime-water; that another pound is dissolved by seven ounces of extra-carbonic acid, so as to form 500 gallons of a solution of bicarbonate of lime; and that the two solutions are mixed, making up together 540 gallons. The nine ounces of caustic lime from the one pound of chalk unite with the seven extra ounces of carbonic acid that hold the other pound of chalk in solution. These nine ounces of caustic lime and seven ounces of carbonic acid form sixteen ounces, that is, one pound of chalk, which, being insoluble in water, becomes visible, at the same time that the other pound of chalk, being deprived of the extra seven ounces of carbonic acid that kept it in solution, reappears. Both pounds of chalk will be found at the bottom after subsidence. The 540 gallons of water will remain above, clear and colourless, without holding in solution any sensible quantity either of caustic lime or of bicarbonate of lime." (pp. 4-5.)

Nothing can be easier than to subject the water in the reservoirs of the different companies to this mode of purification.

In the pamphlet before us it is calculated that the daily supply of water to the metropolis is about 37½ millions of imperial gallons, and that if subjected to the patented process they would deposit no less than 24 tons of solid chalk, making an annual amount of 8000 tons! It is to be recollected that all this solid matter is such as *no filter can separate.*

To say nothing of the probable effects of this purified water on health, (the author does not advert to this subject,) the following are some of its more obvious economical advantages:

1. *The water will be much softened.* Not to mention the advantages hence resulting in regard to cookery, cleanliness, &c. the saving of soap and soda (for washing) will be enormous. This can be shown in the simplest and most striking manner,—*a similar lather being produced in the purified water by about one third of the quantity of soap that is required by the same water before it is purified.* In a word, *very hard water* is instantly converted into *very soft water* by the subtraction of the hardening ingredient, and without the addition of any new ingredient. The author calculates the probable consumption of soap and soda in London at £640,000 per annum, and reckoning the saving from the purified water only at 20 per cent. (a most reasonable estimate) we have here a saving of £128,000. "It is not, however," as he well observes, "alone in soap and soda that a saving arises from the use of soft water in washing. The labour in washing clothes is much increased by the use of hard water, and the wear and tear in consequence is probably a more expensive item than the additional soap."

2. *All Fur in boiling will be prevented*—a most important advantage to those who employ steam, it being well known how all machinery in which boiling water is employed, suffers, in various ways, from the deposition of the earthy matter from water.

3. *Vegetating and colouring matter will be separated.* This is proved by experiment. "The reason is probably twofold: first, the lime-water, which exists in the mixture for a short time as lime-water, may destroy the germs of vegetation; second, the chalk as it forms has a cleansing effect on water, from the property it then possesses of incorporating itself with diffused mud or colouring matter in the water."

4. *The water-insects will be destroyed*, probably by the same means as the vegetation.

The cost of the purifying material is quite insignificant, being little more than that of the burnt chalk required: Mr. Clark calculates this at less than £10 per day for all the water companies of London.

In conclusion, we would express our conviction that this is one of the most valuable applications of science to economical purposes that has been made for a long time, and we think that the inventor has thereby entitled himself to the gratitude of his country.

